# Psoriasis treated with dithranol: a pilot study on in vivo reflectance confocal microscopy

**DOI:** 10.1111/ddg.15825

**Published:** 2025-08-11

**Authors:** Julia K. Winkler, Ferdinand Toberer, Astrid Schirra‐Hoffmann, Lena Vogelgsang, Alexander Enk, Holger A. Haenssle

**Affiliations:** ^1^ Department of Dermatology University of Heidelberg Heidelberg Germany

**Keywords:** Dithranol, psoriasis, reflectance confocal microscopy

## Abstract

**Background:**

There are only limited histomorphological data on the response of psoriatic skin lesions to topical dithranol. In vivo reflectance confocal microscopy (RCM) in psoriatic skin is highly correlated with histopathological findings and allows non‐invasive monitoring of treatment effects on a cellular level.

**Patients and Methods:**

Prospective, single‐center pilot study at a university‐based clinic of dermatology between January 1^st^ and August 30^th^, 2016. Psoriatic lesions of 20 patients receiving dithranol treatment were assessed by RCM at baseline, day 4 and 8 of treatment.

**Results:**

RCM measurements of psoriatic lesions receiving dithranol treatment revealed epidermal histomorphological changes with a strong median reduction of baseline hyperkeratosis by 45.0% (*p* < 0.001), acanthosis by 38.2% (*p* < 0.001), and epidermal thickness by 66.5% (p < 0.001) from baseline until day 8. Moreover, semiquantitative measurements of parakeratosis also showed a significant reduction until day 8 (*p* < 0.001). Correspondingly, RCM revealed dermal histomorphological changes with a decrease in diameter of dermal papillae by 32.1% (*p* < 0.001), decrease in diameter of papillary vessels by 16.9% (*p* = 0.002) and a strong semiquantitative reduction of the inflammatory infiltrate (*p* < 0.001).

**Conclusions:**

Results from our pilot study indicate that topical dithranol treatment of psoriatic lesions may induce a rapid and marked reduction of pathologic epidermal and dermal RCM features.

## INTRODUCTION

Psoriasis vulgaris is a multifactorial, chronic‐inflammatory skin disease with a worldwide prevalence of about 2%.^1^ Psoriatic skin lesions are characterized by well‐demarcated erythematosquamous plaques on the extensor sites of the extremities, the scalp, abdomen, and the sacral region^1^. In clinical routine the diagnosis of psoriasis is made after visual inspection and consideration of the personal and family history. In the setting of clinical trials or in difficult‐to‐diagnose cases a histopathologic examination may be required. The histopathologic hallmarks of psoriasis vulgaris include confluent hyperparakeratosis, regular acanthosis (elongated rete ridges), hypogranulosis (absence or diminished granular layer), thinning of the suprapapillary plate, Munroe microabscesses (collection of neutrophils in the spinous layer), dilated capillaries in dermal papillae, and elongated dermal papillae with a dermal inflammatory infiltrate. Depending on the severity of skin lesions, patient compliance, the presence of psoriatic arthritis, and other comorbidities, physicians may opt for either topical or systemic treatment options. While the range of systemic treatments is broad and continuously expanding, topical corticosteroids, vitamin D_3_ analogues – either as monotherapy or in fixed combination with corticosteroids – and dithranol remain among the most commonly used topical therapies.^2^, ^3^

Dithranol, which has been introduced more than 90 years ago, initiates rapid and strong local reactions accompanied by a marked erythema at the application site.^4^, ^5^ This dose‐dependent skin irritation is a prerequisite for the therapeutic effect on both the hyperproliferative and inflammatory aspects of the psoriasis. At the same time the local erythema with its burning sensation is one of the most limiting side effects.^6^ Despite the excellent therapeutic effects of topical dithranol there are only limited data on its micrometric and morphometric impact on psoriatic skin.^7^ We therefore designed a pilot study addressing the changes in psoriatic skin after repeated application of dithranol. To monitor identical sites within psoriatic lesions, we used non‐invasive In vivo reflectance confocal microscopy (RCM), which provides *en face* imaging along the horizontal plane at cellular resolution. The aim of this pilot study was to assess dynamic changes in psoriasis vulgaris repeatedly treated with topical dithranol by means of RCM imaging.

## PATIENTS AND METHODS

### Patients and study design

The present study was a monocentric, prospective pilot study. It was approved by the local ethics committee and performed in accordance with the declaration of Helsinki principles. All patients gave written informed consent before inclusion. Patients of at least 18 years of age with psoriasis vulgaris and the medical indication for a topical dithranol treatment as inpatients were included between January 1^st^ and August 30^th^, 2016. For each patient one target lesion was specified and imaged by digital dermoscopy and RCM (Vivascope 1500 device, Caliber Imaging & Diagnostics, Rochester, NY, USA) at baseline and day 4 and 8 of dithranol treatment. Target lesions were located on body sites amenable to RCM assessment and exhibited characteristic clinical and dermoscopic features of psoriasis. For reasons of documentation digital dermoscopy images were obtained before each RCM measurement from the exact same localization. Before baseline, all patients were treated with 10% salicylic acid in petrolatum for desquamation. Dithranol 1/16% in a petrolatum base was started once daily (Dithranol 0.0625 g, salicylic acid 3.56 g, white petroleum ad 100 g). Dithranol concentrations were individually escalated (to 0.125%, 0.25%, and 0.5%, respectively) with the aim of maintaining dithranol‐induced local erythema. None of the patients received any other antipsoriatic treatment during and for at least 1 month prior to the study.

### RCM imaging

We used a Vivascope 1500 device (Caliber Imaging & Diagnostics, Rochester, NY, USA) for RCM examinations according to a standardized protocol. Viva Block™ software was used and vertically stacked images were acquired along the z‐axis starting from the epidermal surface at the center of a psoriatic lesion to the dermoepidermal junction zone (DEJ)/upper dermis. The following recordings were assessed, each taken at baseline, day 4 and day 8: *(1)* three z‐stacks from the surface of the stratum corneum to 180 µm depth in a gradation of 4.5 µm, *(2)* four mosaics with an extension of at least 4 × 4 mm at the level of the stratum corneum, stratum granulosum/spinosum, the DEJ and at the level of the papillary dermis. All acquired images were used to systematically assess the presence, extent, and exact measurements in µm of a panel of predefined RCM features (Table 1).

**TABLE 1:** Panel of micrometric and morphometric features determined for each target lesion.

**Histopathologic features**	**Description of criterion in RCM**
Hyperkeratosis in µm	Mean distance out of 3 measurements within target lesion from epidermal surface down to honeycomb pattern along z‐axis
Acanthosis in µm	Mean distance out of 3 measurements within target lesion from honeycomb pattern down to end of rete ridges along z‐axis
Epidermal thickness in µm	Mean distance out of 3 measurements within target lesion from epidermal surface down to end of rete ridges along z‐axis
Parakeratosis (not present, moderate, or strong extent)	Semiquantitative assessment of higher refractile roundish structures within stratum corneum containing bright remnants of keratinocyte's nuclei sometimes surrounded by darker halo Number of nuclei (mean per 5 visual fields, Viva‐Stack 500 × 500 µm) ‐ not present (0 cells) ‐ moderate (1–10 cells) ‐ strong extent (> 10 cells)
Munro microabscesses (not present, moderate, or strong extent)	Semiquantitative assessment of clustered, highly refractile, polymorphonuclear cells at the transition of honeycomb pattern to stratum corneum Number of microabscesses (mean per 5 visual fields) ‐ not present (0) ‐ moderate (1–10) ‐ strong extent (> 10)
Length of dermal papillae in µm	Mean distance out of 3 measurements within target lesion from cobblestone pattern of suprapapillary plate down to end of rete ridges along z‐axis
Diameter of dermal papillae in µm	Mean inner diameter of 3 dermal papillae from 3 measurement sites within target lesion (total of 9 measurements)
Enlarged papillary vessels (not present, moderate, or strong extent)	Semiquantitative assessment of prominent round or linear dark canalicular structures, delimitated by thin walls, within the dermal papillae and at the level of the papillary dermis Number of enlarged vessels > 80 µm (mean per 5 visual fields) ‐ not present (0) ‐ moderate (1–10) ‐ strong extent (> 10)
Diameter of papillary vessels in µm	Mean inner diameter of 3 papillary vessels from 3 measurement sites within target lesion (total of 9 measurements)
Inflammatory infiltrate (not present, moderate, or strong extent)	Semiquantitative assessment of small, homogeneously bright cells consistent with lymphocytes Number of lymphocytes (mean per 5 visual fields) ‐ not present (0) ‐ moderate (1–25) ‐ strong extent (> 25)

### Histopathology

For two patients we performed representative 4‐mm punch biopsies from the localization of each RCM measurement at baseline, day 4 and 8 (three biopsies per patient). Biopsies were taken immediately after RCM measurements from the identical psoriatic patch. Hematoxylin and eosin (HE) stains were obtained. Biopsies were intended for correlation of RCM features with histopathological findings. Histopathology was only performed exemplary in two patients to limit the number of invasive procedures.

### Statistical analysis

Collected data were analyzed by descriptive statistics and provided figures illustrate the results. We used absolute numbers and medians to report and depict RCM measurements. Normalized percentages (baseline medians set to 100%) were used to illustrate median changes over time. A sample size of 20 patients was deemed sufficient to achieve all (descriptive) study goals of this pilot study. Friedmann test was used to investigate for any statistically significant dynamic changes in absolute measurements over time (baseline, day 4, day 8). Results were considered statistically significant at a *p* < 0.05 level. Bonferroni corrections were applied to adjust for multiple comparisons where applicable. All analyses were carried out using SPSS version 29 (IBM, SPSS, Chicago, IL).

## RESULTS

We recruited 20 patients (12 male, 8 female) with a median age of 53.5 years (range 18 to 75 years). At baseline, the median *Psoriasis Area and Severity Index* (PASI) of study participants was 12.9, ranging from 10.1 to 18.0. Psoriatic target lesions for RCM measurements and biopsies were mostly located on the thighs (30%), followed by forearms and lower limbs (25% each). Other anatomic sites were also included, but to a lesser extent (dorsum of hands [10%], abdomen [5%], and back [5%]) (Table 2).

**TABLE 2:** Characteristics of the patients.

	Mean	Range
Median Age	53.5	18‐75
Median PASI	12.9	10.1–18.0
	**n**	**%**
Sex		
Female	8	40.0
Male	12	60.0
Lesion localization		
Trunk	2	10.0
Lower arm	5	25.0
Hand	2	10.0
Upper leg	6	30.0
Lower leg	5	25.0

### Epidermal changes during dithranol treatment

**FIGURE 1:**
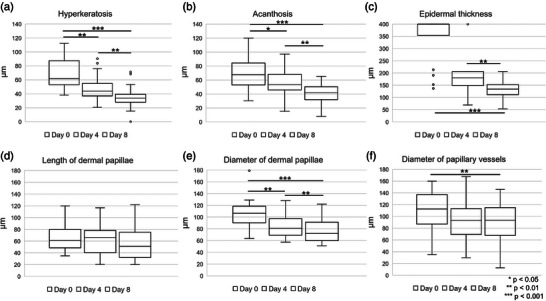
Boxplots depicting (a) hyperkeratosis, (b) acanthosis, (c) epidermal thickness, (d) length of dermal papillae, (e) diameter of dermal papillae, and (f) diameter of papillary vessels at baseline, on day 4, and on day 8 of dithranol therapy. Measurement of (c) epidermal thickness was limited to 400 µm. The upper and lower bounds of boxes indicate the 25th and 75th percentiles, while the median is indicated by the thick line intersecting the box. *p < 0.05; **p < 0.01; ***p < 0.001.

The RCM features of hyperkeratosis, acanthosis, and epidermal thickness showed a significant and pronounced reduction between baseline and day 8 of dithranol treatment. Changes of measurements (µm) over time are depicted in Figure 1a–c. According to our data median hyperkeratosis significantly reduced by 29.1% (p = 0.008) from baseline to day 4, and by 45.0% (*p* < 0.001) from baseline to day 8 (Figure 1a). Similarly, acanthosis decreased by 21.1% (p = 0.014) from baseline to day 4 and by 38.2% (*p* < 0.001) from baseline to day 8 (Figure 1b). The median epidermal thickness was significantly reduced by 66.5% (*p* < 0.001) from baseline to day 8 (Figure 1c). Measurement of maximum epidermal thickness was limited to 400 µm, a value that was reached in 15 patients at baseline and in four patients on day 4. Therefore, the reduction in epidermal thickness under dithranol therapy is likely to have been underestimated.

**FIGURE 2:**
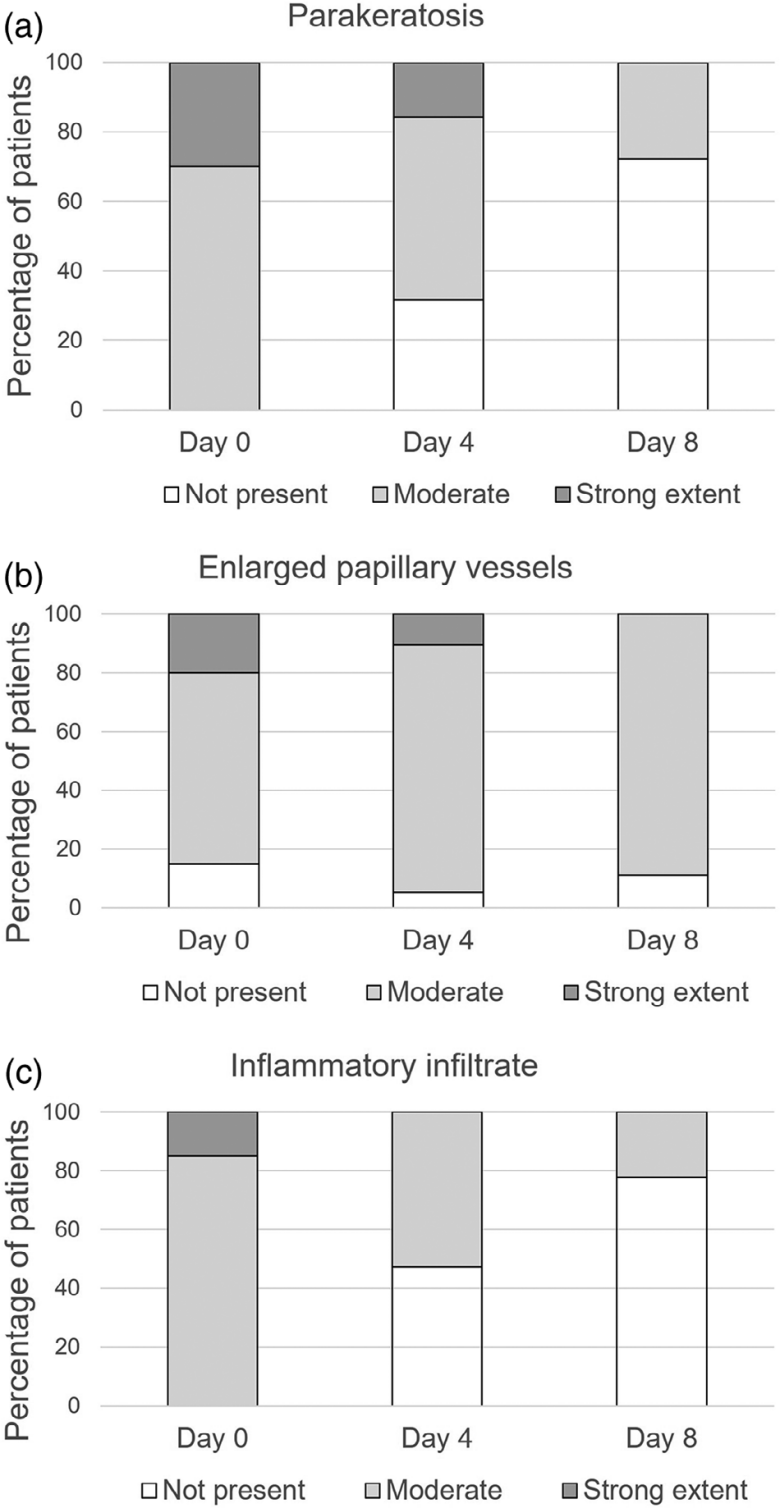
Charts depicting the number of lesions with (a) parakeratosis, (b) enlarged papillary vessels, and (c) inflammatory infiltrate, categorized as absent or present to a moderate or strong extent at baseline, on day 4, and on day 8 of dithranol therapy.

**FIGURE 3:**
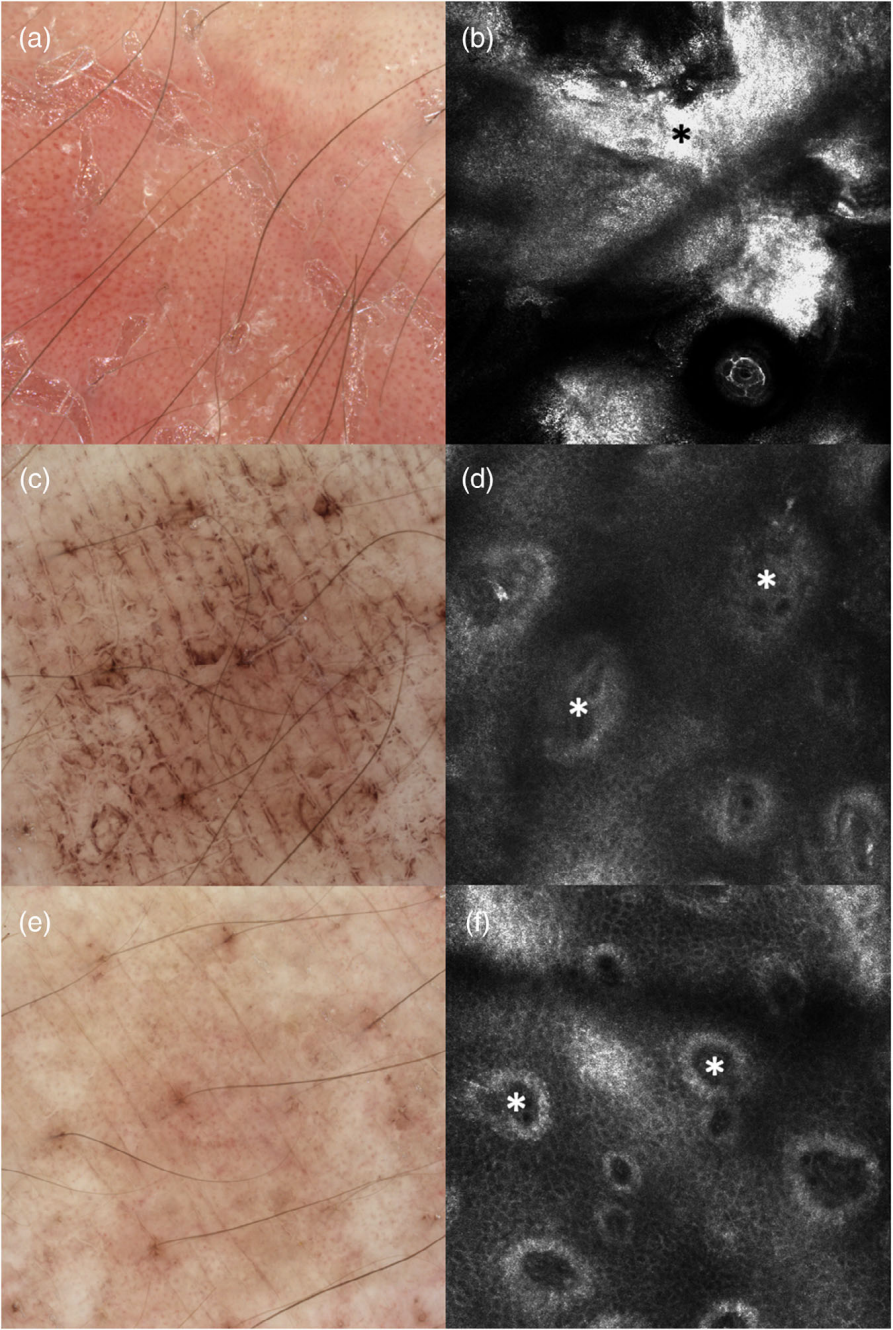
Dermoscopic images and RCM of a psoriatic lesion on the forearm at (a, b) baseline, (c, d) day 4, and (e, f) day 8. (a) Dermoscopy at baseline shows erythema with regularly distributed dotted vessels and white hyperkeratosis, which is replaced by brown discoloration due to dithranol on (c) day 4 and (e) day 8. (b) RCM reveals hyperkeratosis (black star) at baseline, (d) dermal papillae (white stars) with inflammatory cells on day 4, and (f) dermal papillae with smaller diameters (white stars) on day 8.

At baseline parakeratosis with strong extent was present in six patients, with moderate extent in 14 patients. On day 8 of treatment parakeratosis with strong extent was not found in any of the patients, with moderate extent in five patients and not present in 13 patients (Figure 2a). Overall, semiquantitative measurements of parakeratosis showed a significant reduction from baseline until day 8 (*p* < 0.001). Figure 3a, b illustrate a representative target lesion at baseline showing prominent hyperparakeratosis by RCM.

Munro microabscesses with moderate extent were only found in one patient at baseline and another patient on day 4 of dithranol treatment.

### Dermal changes during dithranol treatment

**FIGURE 4:**
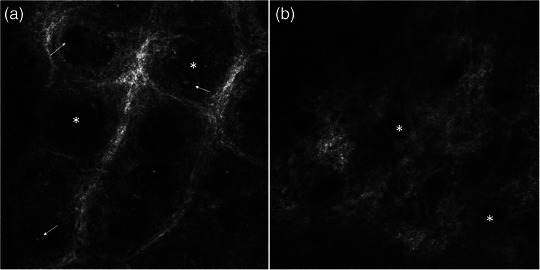
RCM of a psoriatic lesion on the forearm showing (a) extent of inflammatory infiltrate (white arrow) and diameter of dermal papillae (white stars), and (b) their reduction during dithranol therapy.

No significant changes in length of dermal papillae were found (Figure 1d). The median diameter of dermal papillae significantly decreased by 24.3% (p = 0.008) from baseline to day 4 and by 32.1% (*p* < 0.001) from baseline to day 8 (Figure 1e). Figure 4 depicts a representative case with a marked reduction of the diameter of dermal papillae during dithranol treatment. Correspondingly, the median diameter of papillary vessels significantly decreased by 16.9% (p = 0.002) from baseline to day 8 (Figure 1f). At baseline, enlarged papillary vessels with strong extent were present in four patients, with moderate extent in 13 patients, and no enlarged papillary vessels were found in three patients. On day 8, none of the patients showed enlarged papillary vessels with strong extent, 16 with moderate extent and in two patients no enlarged papillary vessels were found. Here, changes from baseline to day 8 were not significant (p = 1.0) (Figure 2b).

At baseline, an inflammatory infiltrate with strong extent was found in three patients and with moderate extent in 17 patients. On day 8, an inflammatory infiltrate with strong extent was detected in none of the patients, moderate infiltrate in four patients and no inflammatory infiltrate in 14 patients (Figure 2c). The extent of the inflammatory infiltrate significantly decreased from baseline until day 4 (p = 0.023) and from baseline until day 8 (*p* < 0.001). A representative reduction of inflammatory cells in the center of papillae as detected by RCM is shown in Figure 3c–f and Figure 4.

### Correlation with histopathology

**FIGURE 5:**
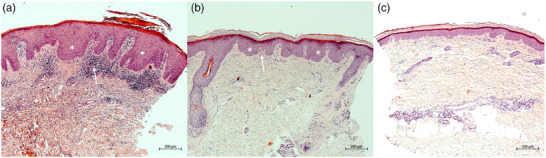
Histopathology of a psoriatic lesion on the forearm at (a) baseline, (b) day 4, and (c) day 8 of dithranol therapy, revealing a marked reduction in hyperparakeratosis, acanthosis (white stars), epidermal thickness, dilated capillaries in dermal papillae, and inflammatory infiltrate (white arrows) (Hematoxylin‐eosin stain, original magnification ×  100).

The exemplary correlation of RCM images with histopathologic slides (HE stains) of target lesions in two patients revealed a high‐level of agreement at all examination times. Biopsies were taken from the identical patch at baseline, day 4 and 8, immediately after performing RCM measurements. Morphological correlation with histopathology confirmed a diminished acanthosis from baseline until day 4 and 8 (Figure 5). Additionally, histopathology revealed the gradual reduction of hyperparakeratosis and the lymphocytic inflammatory infiltrate (Figure 5). Due to the exemplary character of these correlations in only two patients, changes were evaluated only qualitatively, and no quantitative assessment was performed.

## DISCUSSION

The aim of this pilot study was to assess the effect of repeated dithranol applications on aspects of histopathologic features of psoriasis vulgaris by reflectance confocal microscopy, pursuing the dynamic development of each psoriatic target lesion. A strategy to implement RCM for imaging of psoriasis has previously been proposed.^8^ Previous studies have revealed an excellent correlation of histopathologic and RCM features in psoriasis.^9^, ^10^, ^11^, ^12^ This correlation of RCM with histopathology in psoriasis has already been demonstrated under treatment with UVB phototherapy.^13^ According to this study, RCM was considered highly suitable for treatment monitoring in psoriatic disease. Moreover, treatment responses towards systemic (methotrexate, acitretin) or topical treatments (aceclofenac and betamethasone) have also been assessed by RCM.^14^, ^15^

The biochemical basis for the mechanism of action of dithranol is the production of active oxygen species, including singlet oxygen, superoxide anion radicals, and hydroxyl radicals.^7^ Dithranol has an influence on the two major factors which ultimately lead to programmed cell death, such as the activation of caspase and externalization of phosphatidylserine.^1^ Moreover, accumulation of dithranol in keratinocyte mitochondria with subsequent disruption of the mitochondrial membrane potential, cytochrome c release, and caspase‐3 activation was observed.^16^ Therapeutic effects of dithranol have previously been demonstrated in vitro,^1^, ^7^, ^16^ yet our study aimed to assess these In vivo by RCM. A pilot study was performed since this was the first study to assess the effects of dithranol in psoriasis by RCM.

In line with previous studies, we could show that RCM allowed to detect histomorphological correlates of psoriasis such as acanthosis, hyperkeratosis, parakeratosis, dilated papillary vessels and inflammatory infiltrate.^9^ After repeated administration of dithranol RCM showed a reduction in acanthosis. As one of the most successful topical agents for the treatment of psoriasis, dithranol has previously been shown to induce apoptosis in keratinocytes.^1^ We suppose that apoptosis may lead to a reduced number of epidermal keratinocytes and thus a decrease in acanthosis. We could also find a markedly reduced hyperkeratosis and epidermal thickness in RCM images. We hypothesize that this may be related to a normalization of cell division rate during dithranol treatment. Reduction in epidermal thickness has previously been reported as a hallmark of an effective topical treatments in psoriasis.^15^ In our study, dithranol treatment effects on epidermal thickness continuously evolved from baseline until day 8. In our pilot study effects induced by dithranol evolved quite fast, which is in line with previously reported effects in vitro.^1^

In addition to the abovementioned epidermal effects, we found a reduction in the diameter of dermal papillae during dithranol treatment. Additionally, dithranol treatment reduced the diameter of papillary vessels. In contrast, a significant reduction in dilated vessels was not observed in previous studies during treatment with other antipsoriatic agents, such as aceclofenac gel or betamethasone cream.^15^

We also observed a reduction in parakeratosis during dithranol treatment, indicating that dithranol affects epidermal cell differentiation. In our pilot study the inflammatory infiltrate, easily identified by means of RCM,^17^ was reduced during dithranol treatment.

We also assessed Munro microabscesses using RCM during dithranol therapy. However, Munro microabscesses were detected in only one patient at baseline and in another on day 4. Previously, these structures have been reported to occur much more frequently in psoriasis,^18^ which underlines that further studies are needed to either confirm or reject our results. Meanwhile, new dithranol formulations and delivery approaches are being evaluated by means of RCM, enabling visualization of its cutaneous penetration.^19^, ^20^, ^21^, ^22^, ^23^, ^24^

One limitation was the relatively small number of included patients in our pilot study, which still was sufficient to gain important insights showing some marked changes in RCM features during dithranol treatment. Moreover, as a more general limitation of RCM, this technique and its examination results depend on the level of training and experience of the operator. The trained observers assessing RCM images in our study were not blinded for patients and study days. Finally, all listed features assessed by RCM were only studied in psoriatic lesions and not compared with healthy unaffected skin and a specific clinical lesion score for correlation was not assigned.

In summary, our pilot study showed a reduction of pathologic epidermal and dermal features associated with psoriasis during dithranol therapy and found RCM a sufficient tool to assess these features in correlation with histopathology, yet further studies are needed to confirm our findings.

## CONFLICT OF INTEREST STATEMENT

A.H. Haenssle received honoraria and/or travel reimbursements from companies involved in the development of devices for skin cancer screening: SciBase AB, FotoFinder Systems GmbH, Heine Optotechnik GmbH, Magnosco GmbH. J.K. Winkler also received honoraria from FotoFinder Systems GmbH. All other authors declare no conflict of interest.
